# Effects of Solution Treatment on Microstructure Evolution and the Mechanical Properties of GH4780 Superalloy

**DOI:** 10.3390/ma18061288

**Published:** 2025-03-14

**Authors:** Tian-Hao Feng, Xing-Fei Xie, Yang Liu, Jing-Long Qu, Shao-Min Lyu, Jin-Hui Du, Jing-Jing Ruan, Li-Long Zhu

**Affiliations:** 1 Institute for Advanced Studies in Precision Materials, Yantai University, Yantai 264005, China; f17686411107@163.com (T.-H.F.); lyang1537@163.com (Y.L.); ruanjingjingtohoku@163.com (J.-J.R.); 2 Beijing GAONA Materials and Technology Co., Ltd., Beijing 100081, China; qujinglong@cisri.cn (J.-L.Q.); lsmleon@163.com (S.-M.L.); superalloy_1@163.com (J.-H.D.)

**Keywords:** GH4780 superalloy, solution treatment, grain size, mechanical properties, deformation mechanisms

## Abstract

This study systematically investigated the microstructural evolution and mechanical properties of GH4780 superalloy under various solution treatment conditions. Experimental results reveal a strong temperature dependence of grain growth kinetics, with the average grain diameter increasing from approximately 20 μm to 194 μm as the solution temperature rises from 1020 °C to 1110 °C. Mechanical testing demonstrates that grain coarsening reduces the yield strength by 19% at room temperature (from 920 MPa to 743 MPa) and by 9.5% at 760 °C (from 707 MPa to 640 MPa), primarily due to decreased grain boundary density and enhanced dislocation mobility. High-temperature deformation mechanisms were characterized, showing that the reduced grain boundary area facilitates dislocation motion while compromising strength. Furthermore, a grain growth kinetic model was developed, providing a quantitative prediction of microstructural evolution. These findings offer significant guidance for improving the high-temperature performance of GH4780 superalloy by optimizing heat treatment processes.

## 1. Introduction

Ni-based superalloys are extensively utilized in critical aerospace components owing to their high strength and creep rupture lifetime as well as their remarkable resistance to corrosion, oxidation, and fatigue under high-temperature conditions [1,2,3,4]. The hot-section components of modern aero-engines, including turbine blades, nozzle guide vanes, turbine disks, and combustion chambers, are predominantly manufactured from nickel-based superalloys due to their unique combination of high-temperature performance and structural reliability [5,6]. With increasing engine thrust requirements, turbine inlet temperatures continue to rise, demanding corresponding improvements in mechanical properties of superalloys [7]. This technological imperative drives the development of advanced alloy chemistries and processing techniques to enhance high-temperature performance, which is crucial for the evolution of both aviation engines and industrial gas turbines [8]. Within this technological framework, GH4780 superalloy emerges as a prominent nickel-based cast and wrought (C&W) material, demonstrating exceptional mechanical properties and thermal stability at service temperatures of up to 760 °C. These characteristics make GH4780 particularly suitable for demanding high-temperature applications in advanced aerospace and power generation systems.

Heat treatment serves as a critical process for tailoring alloys’ mechanical properties through precise microstructural control, representing the final stage in optimizing material characteristics [9,10]. Proper selection of solution heat treatment temperature enables optimal grain size distribution and precipitation morphology, and therefore the mechanical properties of the alloy can be enhanced [11,12]. Given their fundamental importance, the effects of solution temperature on the microstructure and properties of superalloys have been extensively investigated, with numerous studies focusing on the underlying mechanisms and processing–property relationships. Zhang et al. [13]. investigated the effects of solution treatment temperatures on the microstructure and mechanical properties of Hastelloy X superalloy. The results revealed the temperature-dependent solubility behavior of key precipitated phases, including the γ′ phase and carbides. Optimal solution treatment temperature facilitates the formation of a homogeneous supersaturated solid solution, which is essential for subsequent age hardening. This uniform microstructure enables more consistent and dense precipitation of strengthening phases during aging treatment, ultimately enhancing the mechanical performance of Hastelloy X superalloy.

Li et al. [14] examined the influence of solution treatment on γ′ phase evolution and grain growth kinetics. Their results demonstrated the size-dependent dissolution behavior of γ′ precipitates, with larger particles showing significantly slower dissolution rates. Moreover, γ′ phase dissolution facilitates grain boundary migration, leading to accelerated grain growth with increasing temperature and prolonged holding time. Song et al. [15] studied grain growth behavior in nickel-based superalloys, revealing significant grain boundary-pinning effects from coarse γ′ phases and inert precipitates. Their findings demonstrated distinct grain growth mechanisms under different thermal conditions; at sub-solvus temperatures, grain size is primarily controlled by the size and volume fraction of γ′ precipitates, and at super-solvus temperatures, grain growth is restricted by the thermal stability of MC carbides and yttrium oxides. Complete grain growth observed at 1270 °C was attributed to the coarsening and dissolution of MC carbides and yttrium oxides, which subsequently lost their grain boundary-pinning capability. Zhou et al. [16] systematically investigated the effect of solution temperature on the microstructural evolution and creep behavior of Ni-Co-based superalloys for turbine disk applications. The results showed that microstructural variations following different solution treatments were primarily governed by the distribution of the γ′ phase and the morphological characteristics of grain boundary carbides. As the solution temperature increased, rapid dissolution of primary γ′ phase was observed, accompanied by increased volume fractions of secondary and tertiary γ′ precipitates. This microstructural transformation significantly enhanced the alloy’s mechanical properties, particularly its creep resistance.

As a newly developed Ni-based C&W superalloy, the microstructural evolution mechanisms and property optimization of GH4780 superalloy during heat treatment remain insufficiently understood. The grain size of the GH4780 superalloy can be precisely controlled through the synergistic optimization of temperature–time parameters during solution treatment. This control directly influences critical properties, including strength, plasticity, and creep resistance. This mechanism is fundamentally rooted in balancing the requirements of grain refinement strengthening and high-temperature stability, thereby providing a reliable material foundation for extreme environmental applications, such as aerospace engineering.

In this study, the newly developed GH4780 superalloy by Beijing GAONA Materials and Technology Co., Ltd. (Beijing, China), was thoroughly investigated, focusing on the influence of solution temperature (1020~1110 °C) on its microstructure and mechanical properties. Special emphasis was placed on achieving precise grain size control through the optimization of solution treatment parameters to enhance its mechanical performance. A temperature-dependent grain growth model incorporating MC carbide-pinning effects was uniquely established, representing a critical advancement due to the 1080 °C threshold for rapid coarsening observed in GH4780 superalloy. This behavior fundamentally differs from conventional Ni-Co alloys, which exhibit gradual coarsening above 1050 °C, providing critical theoretical insights for optimizing heat treatment processes. These findings will offer significant contributions to the mission to meet the stringent high-temperature performance requirements of the components of advanced aircraft engines’ hot sections.

## 2. Experimental

### 2.1. Alloy Preparation and Heat Treatment

In this study, GH4780 superalloy ingots were initially processed in a ZG-0.5LB vacuum induction furnace (Jinzhou Oriental Gold Technology Research Co., Ltd., Jinzhou, China). After homogenization, the ingots underwent thermomechanical processing, commencing with precision forging utilizing a 1-ton electro-hydraulic forging hammer (Jier Machine-Tool Group Co., Ltd., Jinan, China), followed by controlled hot-rolling operations performed on a 280-model horizontal rolling mill (Yantai Haige Machine Tools Co., Ltd., Yantai, China). The actual chemical composition of the alloy was measured and is detailed in Table 1. The uneven ends of the rolled bars were machined using wire electrical discharge machining (Suzhou Sanguang Science & Technology Co., Ltd., Suzhou, China), and then the specimens were cut into specimens with specific dimensions of Ø19 × 70 mm and Ø19 × 20 mm. Subsequently, the samples underwent heat treatment according to the procedure detailed in Table 2. For samples AT1~AT4, air cooling (AC) using a fan was performed after solution and aging heat treatments. Additionally, samples ST1~ST4 were water-quenched (WQ) immediately after solution heat treatment to investigate the microstructural evolution of the alloy.

### 2.2. Mechanical Tests

**Figure 1:**
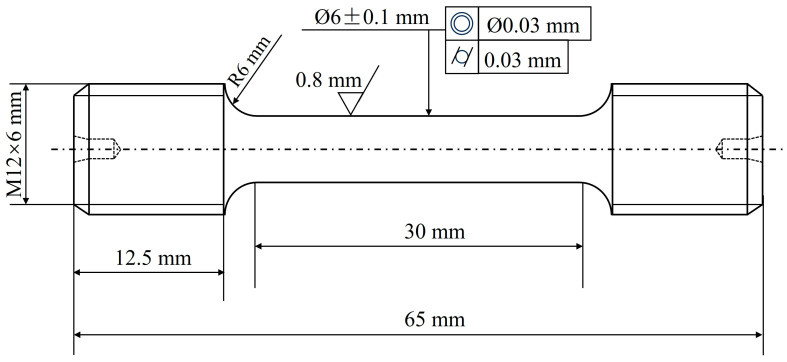
Schematic diagram of the GH4780 specimens for tensile testing.

The samples AT1~AT4 were subjected to tensile tests at both room temperature and the evaluated temperatures in two sets of parallel experiments. Tensile tests were conducted at room temperature in accordance with the ASTM E8 standard [17] and at high temperature (760 °C) in accordance with the ASTM E21 standard [18], using machined cylindrical threaded dogbone specimens with a gauge length of 65 mm and a gauge diameter of 6 mm, as schematically illustrated in Figure 1. ASTM E8 (room temperature) and ASTM E21 (high temperature) were selected to align with industry standards for aerospace materials, ensuring comparability with existing datasets and certification requirements.

### 2.3. Microstructural Characterization

The microstructure and phase compositions of the alloy specimens before and after mechanical testing were characterized using an optical microscope (OM, Olympus Corporation, Tokyo, Japan), electron probe micro-analyzer (EPMA, JEOL Ltd., Tokyo, Japan), scanning electron microscope (SEM, JSM-7200F from JEOL Ltd., Tokyo, Japan) equipped with an energy-dispersive spectroscope (EDS in JSM7200F), electron backscatter diffraction (EBSD in JSM7200F), and a transmission electron microscope (TEM). SEM-EDS elemental mapping elucidated carbide distributions, EBSD analysis with kernel average misorientation (KAM) mapping quantified grain boundary characteristics, and TEM imaging revealed stacking faults within M_23_C_6_ carbides. This multiscale analytical approach directly linked critical microstructural features (e.g., γ′ precipitates) to mechanical performance. Firstly, metallographic preparation of the samples was performed, which included step-by-step grinding with abrasive paper, followed by mechanical polishing and finally etching in a corrosive solution for approximately 4 min. This corrosive solution contained 1.5 g of CuSO_4_ (Wanhua Chemical Group Co., Ltd., Yantai, China). 20 mL of C_2_H_6_O (Sinopharm Chemical Reagent Co., Ltd., Shanghai, China), and 20 mL of HCl (Sinopharm Chemical Reagent Co., Ltd., Shanghai, China). For EBSD analysis, samples were extracted from the longitudinally sectioned fracture surfaces and then subjected to electrolytic polishing in a solution of 80 mL CH_3_OH (Sinopharm Chemical Reagent Co., Ltd., Shanghai, China) and 10 mL H_2_SO_4_ (Sinopharm Chemical Reagent Co., Ltd., Shanghai, China) using a voltage of 20 V for 5~6 s. For the preparation of SEM samples, an electrochemical etching technique was employed to selectively remove the γ matrix, allowing for a clearer examination of the features of γ′ precipitates. The electrochemical etching was conducted at a voltage of 4.5 V with a duration of 4~6 s in a solution consisting of 15 g Cr_2_O_3_ (Shanghai Liantian Material Technology Co., Ltd., Shanghai, China), 10 mL H_2_SO_4_, and 150 mL H_3_PO_4_ (Sinopharm Chemical Reagent Co., Ltd., Shanghai, China). TEM and STEM analyses were performed using an FEI-Talos F200X microscope (Thermo Fisher Scientific Inc., Hillsboro, OR, USA) equipped with an advanced super-XTM EDS detector. For the preparation of electron transparent disks intended for TEM analysis, a Struers TenuPol-3 twin-jet electro-polisher (Struers A/S, Ballerup, Denmark) was employed. The electrolyte solution consisting of 90% CH_3_OH and 10% HClO_4_ (Sinopharm Chemical Reagent Co., Ltd., Shanghai, China) was applied at a temperature of −30 °C with an operating voltage of 20 V.

## 3. Results and Analysis

### 3.1. Microstructure of GH4780 Superalloy

**Figure 2:**
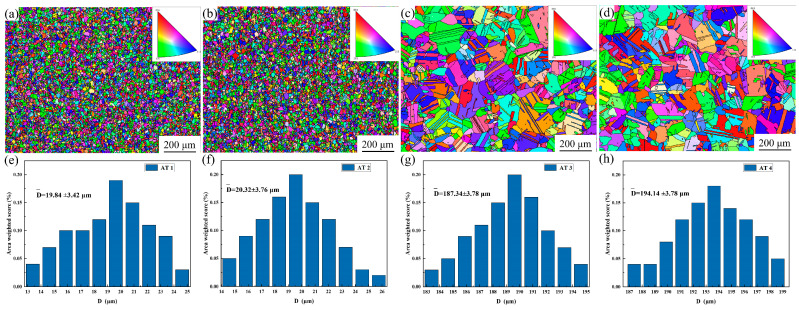
EBSD IPF maps and grain size distribution plots of GH4780 superalloys after different heat treatments: (**a**,**e**) AT1; (**b**,**f**) AT2; (**c**,**g**) AT3; (**d**,**h**) AT4.

Figure 2a–d display EBSD inverse pole figure (IPF) maps of GH4780 superalloys after different solution and aging heat treatments, while the grain size distribution is depicted in Figure 2e–f. It can be observed that the grain size of the alloy remained at approximately 20 µm and did not exhibit significant changes when solution heat treatment was conducted at temperatures of 1020 °C and 1050 °C. When the temperature was increased to 1080 °C and 1110 °C, the average grain size rapidly increased to approximately 187 μm and 194 μm, respectively.

**Figure 3:**
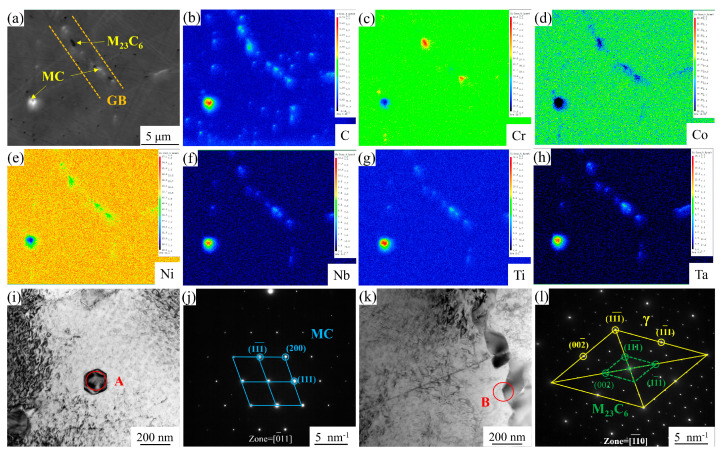
Composition and crystal structure analyses for the MC and M_23_C_6_ carbides in the AT1 alloy: (**a**) BSE image highlighting the location and morphology of the carbides; (**b**–**h**) elemental distributions in the carbides; (**i**) TEM bright-field (BF) image and (**j**) obtained SAED patterns of the MC carbide from the region A in (**i**); (**k**) TEM BF image and (**l**) obtained SAED patterns of the M_23_C_6_ carbide from the region B in (**k**).

Figure 3a shows the backscattered electron diffraction (BSE) image of the carbides in the AT1 alloy, where coarse carbides are uniformly dispersed throughout the alloy, while fine carbides are located at the grain boundaries. The composition distribution of these carbides was mapped using EPMA, as shown in Figure 3b–l. The coarse carbides were found to be enriched in C, Nb, Ti, and Ta. Their crystal structure was determined to be face-centered cubic (FCC) through TEM selected-area electron diffraction (SAED) analysis, as shown in Figure 3i,j. As a result, these coarse carbides can be identified as MC carbides. The finer carbides at the grain boundaries were found to be rich in C and Cr. These carbides also exhibited an FCC structure but maintained a specific orientation relationship with the γ matrix substrate, Figure 3k,l. The calculated lattice constant of this carbide is 10.66 Å, which is approximately three times the matrix lattice constant of 3.6 Å. Therefore, the fine precipitated phase at the grain boundary can be identified as M_23_C_6_ carbides.

**Figure 4:**
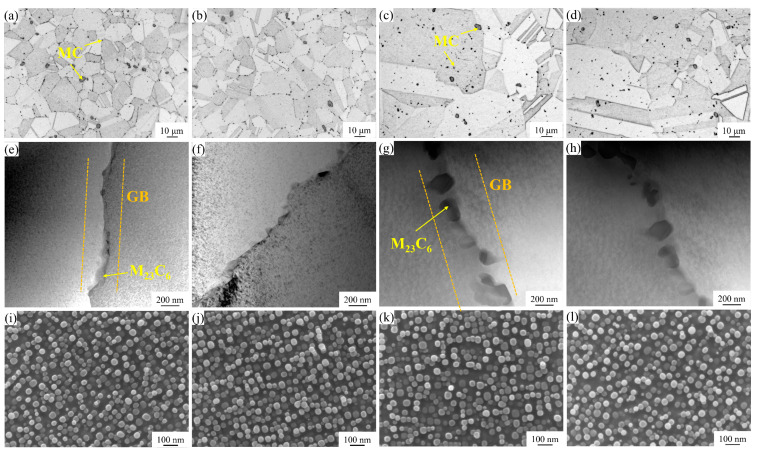
The microstructure of the GH4780 superalloy after various heat treatments, illustrating the precipitation of carbides and secondary γ′ phases: (**a**–**d**) OM images showing the MC carbides in the ST1~ST4 alloys; (**e**–**h**) TEM HAADF images showing the M_23_C_6_ carbides in the AT1~AT4 alloys; (**i**–**l**) SEM images showing the fine secondary γ′ phase in the AT1~AT4 alloys.

Figure 4a–d present the OM images of MC carbides in solution-treated GH4780 superalloy, demonstrating negligible variations in both size distribution and morphological characteristics among the ST1 to ST4 alloy conditions. Figure 4e–h show the TEM high-angle annular dark-field (HAADF) images of M_23_C_6_ carbides in the aged specimens of AT1 to AT4, illustrating a pronounced temperature-dependent growth behavior. Specifically, both the size and morphological complexity of M_23_C_6_ carbides exhibit substantial enhancement in the AT3 and AT4 alloys as the solution temperature increases. In the GH4780 superalloy, M_23_C_6_ carbides nucleate primarily at grain boundaries, and as the solution temperature increases, the grain size enlarges, leading to a reduction in the number and density of the grain boundaries. Consequently, the M_23_C_6_ carbides that form become larger and exhibit more complex morphologies due to the decreased frequency of nucleation sites. SEM images of fine spherical secondary γ′ precipitates distributed within the grains are shown in Figure 4i–l. The size distribution, morphological sphericity, and area fraction of the secondary γ′ precipitates in the aged state after different solution heat treatments are essentially uniform across the four superalloys AT1~AT4, with an average particle size and area fraction of approximately 30 nm and 23.8%, respectively. These M_23_C_6_ carbides and secondary γ′ phases nucleate and grow during the aging heat treatment at 800 °C for 8 h [19,20].

### 3.2. Tensile Properties of GH4780 Superalloy

**Figure 5:**
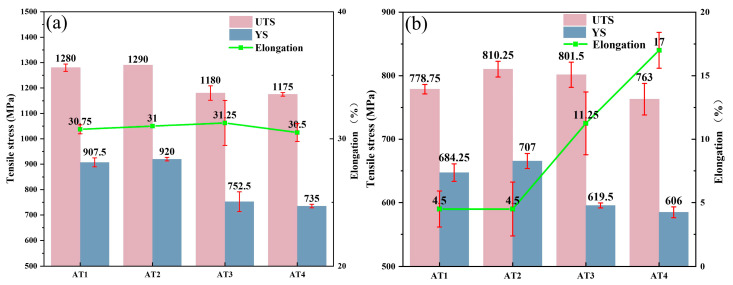
Tensile properties of the GH4780 superalloys tested at (**a**) room temperature and (**b**) a high temperature of 760 °C.

The tensile properties of GH4780 superalloys, including yield strength (YS), ultimate tensile strength (UTS), and elongation, are systematically presented in Figure 5. Comparative analysis reveals that the AT1 and AT2 alloys exhibit nearly identical mechanical performance, with both demonstrating superior YS and UTS values compared to the AT3 and AT4 alloys across all testing temperatures. Notably, the AT2 alloy achieves optimal strength characteristics, with peak values of 920 MPa YS and 1290 MPa UTS at room temperature. When subjected to elevated temperature conditions, it exhibits only a 23.2% reduction in YS (from 920 MPa to 707 MPa) and retains 37.2% of its UST (from 1290 MPa to 810 MPa), demonstrating exceptional thermal stability. In terms of ductility, the AT4 alloy demonstrates exceptional elongation properties, particularly at high temperature, where it reaches a maximum elongation of 17%, significantly exceeding that of other tested alloys.

**Figure 6:**
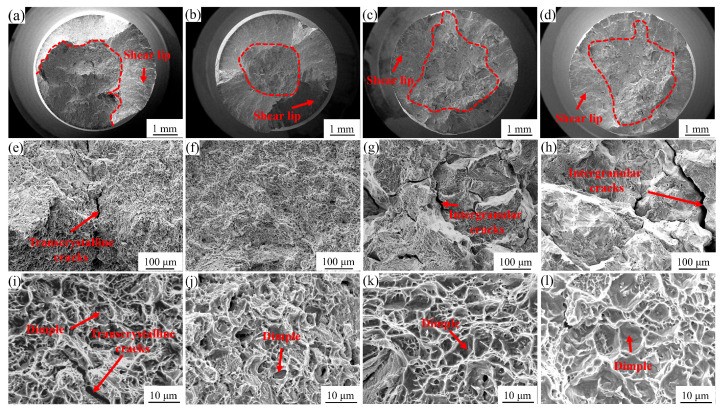
Fracture morphology of the GH4780 superalloys after tensile testing at room temperature: (**a**–**d**) macroscopic fracture surfaces of the AT1~AT4 alloys; (**e**–**l**) high-magnification SEM images of the microscopic fracture morphology in the AT1~AT4 alloys, revealing detailed features of dimple structures and fracture mechanisms.

Figure 6 illustrates the fracture characteristics of the GH4780 superalloys after room-temperature tensile testing. Figure 6a,b depict the macroscopic fracture morphology of the AT1 and AT2 alloys, exhibiting a mixed fracture mode consisting of transcrystalline rupture and ductile fracture features. Figure 6c,d present the macroscopic fracture morphology of the AT3 and AT4 alloys, revealing a combination of intergranular fracture and ductile fracture patterns. At the microscopic level, Figure 6e,f,i,j show the detailed fracture surfaces of the AT1 and AT2 alloys, characterized by transcrystalline fracture mechanisms and well-defined dimple morphologies, indicative of their ductile behavior. In contrast, Figure 6g,h,k,l display the microscopic fracture morphologies of the AT3 and AT4 alloys, dominated by intergranular fracture features accompanied by dimple structures.

The distinct fracture behaviors observed in the AT1/AT2 and AT3/AT4 alloys highlight the significant influence of solution temperature on the microstructural characteristics and mechanical performance of GH4780 superalloys. The observed variation in fracture modes among the alloys subjected to different solution temperatures can be attributed to microstructural evolution, particularly grain growth, which reduces the density of grain boundaries. This microstructural change promotes dislocation accumulation at the remaining grain boundaries, leading to localized stress concentration. Under tensile loading, crack initiation preferentially occurs in these stress-concentrated regions, followed by propagation along the grain boundaries, ultimately resulting in intergranular fracture.

**Figure 7:**
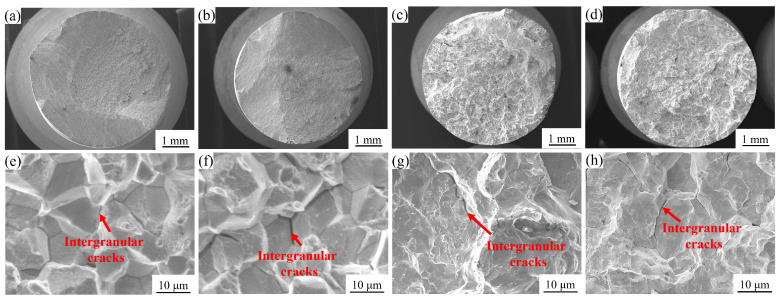
Fracture morphology of the GH4780 superalloy after tensile testing at a high temperature of 760 °C: (**a**–**d**) macroscopic fracture surfaces of the AT1~AT4 alloys; (**e**–**h**) high-magnification SEM images of the microscopic fracture morphology in the AT1~AT4 alloys.

Under high-temperature tensile testing, the AT1~AT4 alloys predominantly exhibit intergranular fracture as the primary failure mode. As shown in Figure 7, the macroscopic fracture surfaces display clear intergranular fracture characteristics, while microscopic analysis reveals a distinct rock candy-like morphology along the grain boundaries, as illustrated in Figure 7e–h. This fracture behavior is consistent with the well-documented phenomenon in superalloys wherein elevated temperatures significantly reduce grain boundary strength, promoting crack propagation along these weakened boundaries during deformation [21].

**Figure 8:**
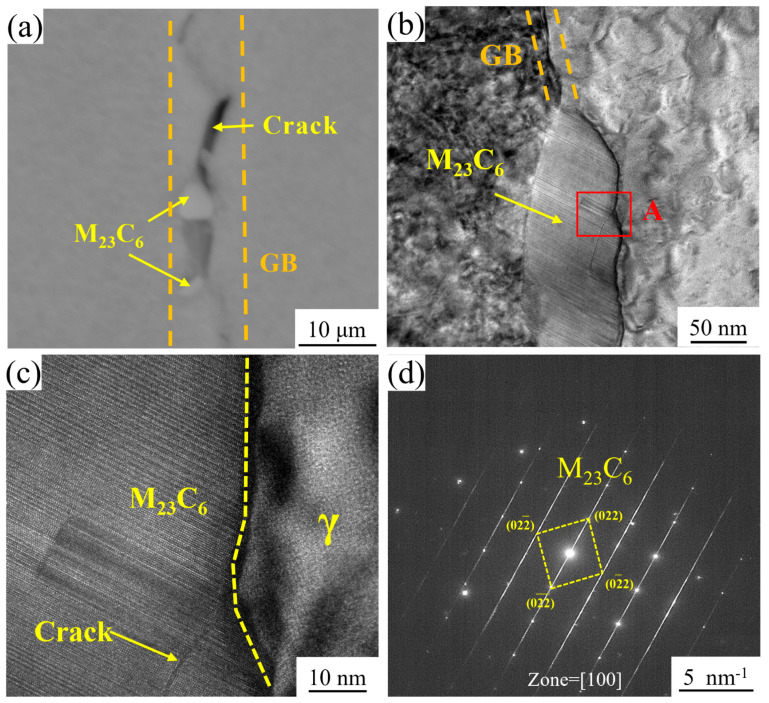
TEM analysis of crack initiation mechanisms associated with M_23_C_6_ carbides: (**a**) crack propagation behavior adjacent to M_23_C_6_ carbides at grain boundaries; (**b**) formation of stacking faults (SFs) within M_23_C_6_ carbides; (**c**) magnified view of region A from (**b**), demonstrating crack nucleation and growth within M_23_C_6_ carbides; (**d**) corresponding Fourier transform pattern of (**c**), showing distinct diffraction spots that confirm the crystallographic features of the cracked carbide structure.

The enhanced elongation observed in AT3 and AT4 alloys, as shown in Figure 5b, can be attributed to microstructural modifications resulting from increased solution temperatures, particularly grain growth, which reduces the density of grain boundaries. However, this microstructural evolution also facilitates the precipitation of M_23_C_6_ carbides at grain boundaries, which usually has a detrimental effect on mechanical properties. As demonstrated in Figure 8a, the aggregation and coarsening of M_23_C_6_ carbides create localized stress concentrations, serving as preferential sites for crack initiation during high-temperature deformation. This mechanism significantly reduces the plastic deformation capability of the matrix, thereby reducing the yield strength of the alloy. Furthermore, Figure 8b–d reveal the formation of extensive stacking faults within grain boundary M_23_C_6_ carbides during deformation. This structural instability leads to carbide softening at elevated temperatures, promoting crack nucleation within the carbides themselves. The combined effects of carbide softening and stress concentration at grain boundaries severely compromise boundary integrity, ultimately leading to a substantial reduction in the overall strength of the superalloy.

## 4. Discussion

### 4.1. Effect of Solution Treatment on the Microstructural Evolution of GH4780 Superalloy

The above experimental results demonstrate a strong correlation between solution temperature and grain size evolution in GH4780 superalloy, which directly governs its mechanical performance. As shown in Figure 2, the GH4780 superalloy undergoes a distinct grain growth transition within the critical temperature range of 1050~1080 °C. Notably, when the solution temperature exceeds 1080 °C, a marked acceleration in grain growth kinetics is observed during thermal processing. Therefore, this paper intends to establish a comprehensive kinetic model to describe the grain growth behavior of the alloy during solution treatment, focusing on the temperature range of 1080~1110 °C. The Sellars model, originally developed for predicting austenite grain growth kinetics, was adapted and implemented to quantitatively characterize the grain size evolution of GH4780 superalloy under various solution treatment conditions [22]:

(1) $$D^{n}=D_{0}+Atexp(-\frac{Q}{RT})$$

In Equation (1), the parameters are defined as follows. *D* denotes the final average grain diameter (μm), *D*_0_ represents the initial grain size prior to solution treatment (μm), *t* corresponds to the isothermal holding time (s), T indicates the solution temperature (K), Q stands for the activation energy of grain boundary migration (J/mol), and R is the universal gas constant (8.314 J·mol^−1^·K^−1^). Experimental observations reveal that grain growth behavior within the temperature range of 1050~1110 °C demonstrates size-independent characteristics, allowing for the elimination of the initial grain size term *D*_0_. Consequently, the Sellars model can be simplified to the following form, as shown in Equation (2):

(2) $$D^{n}=Atexp(-\frac{Q}{RT})$$

Taking the logarithm on both sides of the simplified Sellars model yields Equation (3):

(3) $$\ln D=\frac{1}{n}lnA+\frac{1}{n}lnt-\frac{Q}{nR}\frac{1}{T}$$

**Figure 9:**
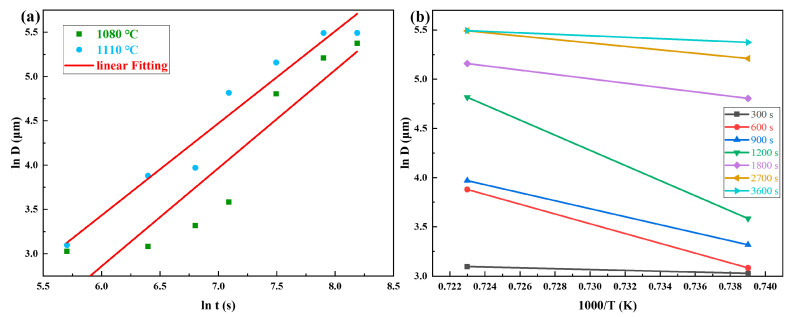
Quantitative analysis of grain growth kinetics using the Sellars model: (**a**) ln D − ln t curves for the index n; (**b**) ln D − 1000/T curves for the activation energy Q.

Based on the experimental data, the relationship curves between ln *D* − ln *t* and between ln *D* − 1000/*T* were acquired, as depicted in Figure 9a,b, respectively. By linear fitting, the values of *n* = 0.99, *Q* = 256,978.26 J·mol^−1^, and *A* = 4.03 × 10^8^ were calculated. Substituting these parameters into Equation (2), the grain growth equation for GH4780 superalloy under different solution temperatures and holding times can be obtained, as shown in Equation (4):

(4) $$D^{0.99}=4.03\times 10^{8}\cdot t\cdot exp(-\frac{256,978.26}{8.314T})$$

The good agreement presented in Figure 9 clearly demonstrates that this equation is capable of accurately depicting the trend of variation in grain size in the GH4780 superalloy throughout the solution treatment process.

Moreover, the microstructures of the superalloys at different solution temperatures were analyzed to explore the impact of microstructure on grain growth. Grain growth represents a microstructural evolution process propelled by grain boundary migration, which commences with a decrease in surface energy. As the solution temperature increases, the thermal energy of atoms increases, causing an elevation in their vibrational amplitude. This increased thermal energy promotes thermally activated atomic motion and boosts atomic diffusion. As a result, the diffusion of atoms in the vicinity of grain boundaries is enhanced, making it more probable that these atoms transfer into adjacent grains. This transfer leads to the gradual coalescence and growth of the grains [23,24]. Furthermore, at high temperatures, atoms at grain boundaries possess higher energies, enabling them to more readily surmount energy barriers and migrate along the grain boundaries. This migration of grain boundaries causes the interface between adjacent grains to gradually disappear, thereby resulting in an increase in grain size [25].

**Figure 10:**
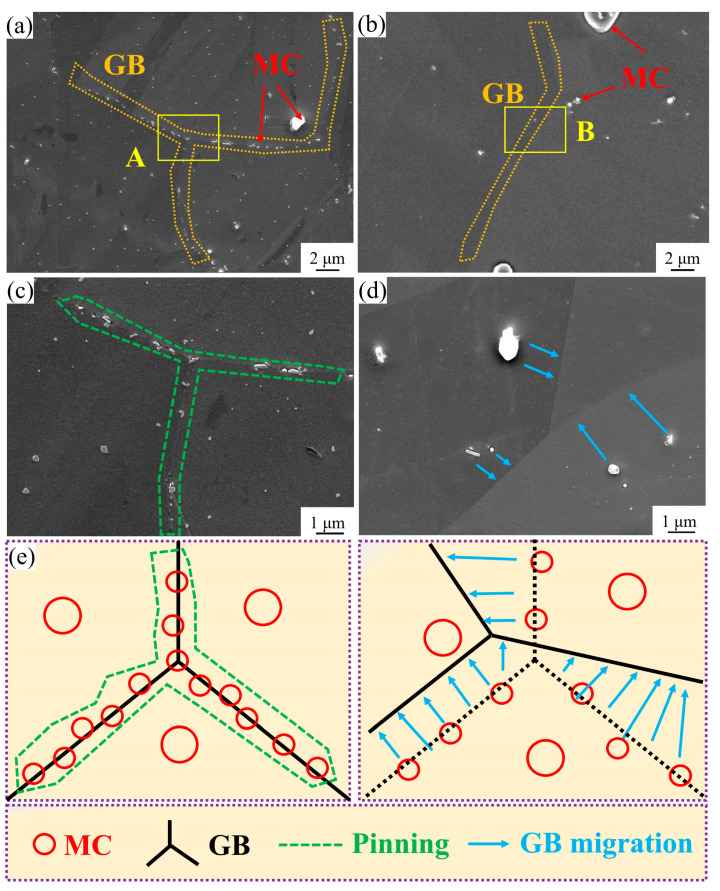
Schematic illustration of grain boundary migration mechanisms: (**a**) pinning effect of MC carbides on grain boundary motion in ST1 alloy; (**b**) unrestricted grain boundary migration in ST3 alloy; (**c**) and (**d**) magnified views of regions A and B in (**a**) and (**b**), respectively; (**e**) proposed mechanism of temperature-dependent grain boundary migration.

Furthermore, the second-phase particles present in the superalloy can act as obstacles to the migration of grain boundaries, impeding the movement of grain boundaries and thus preventing the growth grains [26,27]. As is evident in Figure 10a,c for the ST1 and ST2 alloys, the grain boundaries are pinned by MC carbides, which in turn inhibits grain growth. When the solution temperature is increased, grain boundaries begin to migrate, as observed in Figure 10b,d for the ST3 and ST4 alloys. In these alloys, the grain boundaries are no longer under the pinning influence of MC carbides, thereby enabling the process of grain growth to take place. A schematic diagram that vividly illustrates the migration of grain boundaries with the increase in solution temperature is presented in Figure 10e.

### 4.2. Effect of Grain Size on Tensile Properties of GH4780 Superalloy at Room Temperature

Grain size significantly influences the tensile properties of the GH4780 superalloy. The relationship between the grain size and the yield strength of the alloy follows the Hall–Petch relationship, as described by Equation (5) [28]:

(5) $$\sigma_{S}={\sigma_{0}+kd}^{-\frac{1}{2}}$$

**Figure 11:**
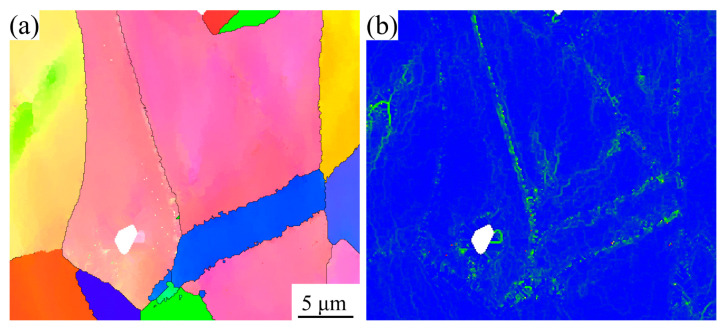
EBSD images showing the grains and dislocation densities in the AT3 alloy after room-temperature tensile testing: (**a**) IPF map; (**b**) KAM map.

In this equation, *σ_s_* stands for the yield strength of the alloy, *k* is the proportional coefficient known as the Hall–Petch constant, *d* denotes the average grain size, and *σ_0_* represents the intrinsic lattice friction stress of the alloy. The experimental results demonstrate a clear correlation between solution temperature and grain size evolution, where elevated temperatures promote grain coarsening, consequently reducing the yield strength of the alloy, as quantitatively illustrated in Figure 5a. At room temperature, grain boundaries exhibit superior mechanical strength compared to intragranular regions, thereby playing a crucial role in the strengthening mechanisms of GH4780 superalloy. A fine grain size gives rise to a higher density of grain boundaries that effectively impedes the motion of dislocations during the deformation process, resulting in enhanced yield strength and tensile strength [29,30,31]. This mechanism is further supported by EBSD IPF and KAM measurements, as presented in Figure 11, which reveal a high dislocation density at the grain boundary in the AT3 alloy. However, it should be noted that while grain refinement strengthens the alloy, it simultaneously increases the defect density. Conversely, grain coarsening reduces the grain boundary area, thereby decreasing defect concentration and improving plastic deformation capability [32]. Therefore, among the studied alloys, the AT4 alloy demonstrates superior plasticity.

### 4.3. Effect of Microstructure on High-Temperature Tensile Properties of GH4780 Superalloy

The tensile properties of the GH4780 superalloy at high temperatures are inherently associated with its resistance to crack initiation and propagation. During the deformation process, the density of geometrically necessary dislocations in the fine-grained regions is significantly higher than that in the coarse-grained regions. At high temperatures, grain boundaries are in a more viscous state, making them more susceptible to sliding and migration under the influence of external forces [33,34]. This thermally activated process diminishes the strengthening effect typically associated with grain refinement, suggesting that controlled grain coarsening may effectively mitigate grain boundary sliding and potentially enhance high-temperature mechanical performance.

**Figure 12:**
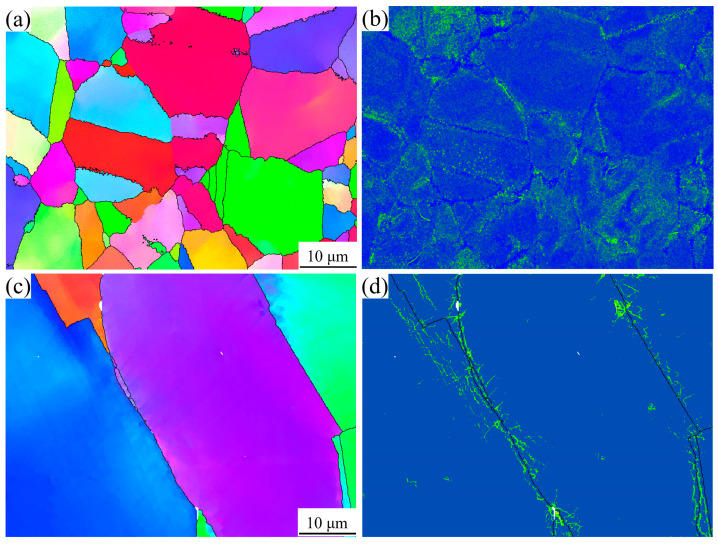
EBSD images showing the grains and dislocation densities in the AT1 and AT3 alloys after high-temperature tensile testing: IPF maps for (**a**) the AT1 alloy and (**c**) the AT3 alloy; KAM maps for (**b**) the AT1 alloy and (**d**) the AT3 alloy.

Experimental data demonstrate distinct reductions in yield strength at high temperatures, of approximately 24% for the AT1 and AT2 alloys and 17% for the AT3 and AT4 alloys, when compared to the values at room temperature. This differential response can be attributed to the more uniform deformation behavior of coarse-grained structures at high temperatures, where homogeneous dislocation motion reduces localized stress concentrations and minimizes damage accumulation. This enhanced high-temperature performance is further supported by the EBSD KAM analysis in Figure 12, which compares the dislocation distributions in the AT1 and AT3 alloys after high-temperature tensile testing. The KAM maps clearly indicate higher dislocation densities in fine-grained regions, consistent with the observed mechanical behavior.

**Figure 13:**
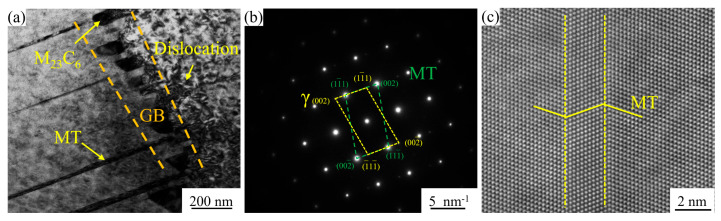
Microstructural characterization of MT interactions with grain boundaries in the AT3 alloy: (**a**) TEM BF image demonstrating MT inhibition at grain boundaries; (**b**) SAED pattern of MTs; (**c**) high-resolution TEM (HRTEM) image revealing MT morphology and atomic structure.

Under high-temperature service conditions, superalloys exhibit distinct deformation mechanisms that significantly differ from room-temperature behavior, making the investigation of these mechanisms essential for performance optimization of GH4780 superalloy. Microtwins (MTs) are one of the primary deformation mechanisms during high-temperature treatment, and their formation is closely related to the crystal structure of the superalloy. In the FCC structure, the layered atomic arrangement enables relative slip between atomic layers through twin boundaries, resulting in the formation of MTs. During alloy plastic deformation, dislocation motion and interactions serve as the main driving forces for MT formation. When external stress is applied, dislocations slip and climb within the crystal. Under specific conditions, dislocation accumulation can induce atomic layer slip, forming micro-twin boundaries and creating MTs. However, MT propagation may cause stress concentration during deformation, leading to crack initiation and strength degradation. Excessive MT formation can increase alloy brittleness, as micro-twin boundaries may provide paths for crack propagation, ultimately reducing the performance of the superalloy [35,36,37]. As shown in Figure 13, TEM bright-field (BF) images reveal the synergistic effect of grain boundaries in hindering MT propagation. During deformation, MT expansion is effectively constrained by the high density of grain boundaries in the fine-grained AT1 and AT2 alloys, thereby enhancing their strength.

## 5. Conclusions

The present study systematically investigated the effects of solution temperature on the microstructural evolution and mechanical properties of GH4780 superalloy. Through comprehensive microstructural characterization and mechanical testing, the following conclusions could be drawn.

(1) In GH4780 superalloy, grain size evolution strongly depends on solution treatment temperatures. MC carbides are crucial for pinning grain boundaries, effectively restraining grain growth below 1080 °C. However, above 1080 °C, thermally activated grain boundary migration significantly accelerates grain growth kinetics. Furthermore, a grain growth kinetic model has been established within the temperature range of 1080~1110 °C.

(2) The mechanical properties of GH4780 superalloy demonstrate a significant grain size dependence. The AT2 alloy, with an average grain size of 20 µm, exhibits superior room-temperature yield strength of 920 MPa and tensile strength of 1290 MPa. This strength advantage is maintained at elevated temperatures, demonstrating yield strength of 707 MPa and tensile strength of 810 MPa at 760 °C. In contrast, the coarse-grained AT4 alloy (average grain size ≈ 194 µm) shows enhanced ductility, achieving maximum elongation of 17% at 760 °C.

(3) High-temperature tensile loading induces grain boundary weakening in GH4780 superalloy, primarily causing intergranular fracture. This process is accelerated by M_23_C_6_ carbides at grain boundaries, where stress concentrations initiate cracks that propagate along boundary networks. During deformation, dislocation interactions generate microtwins (MTs) that reduce strength when propagating. However, grain boundaries effectively hinder MT expansion, enhancing alloy strength through a pinning mechanism, thereby strengthening the alloy.

(4) The grain growth kinetics model and mechanical property data established in this study provide a comprehensive quantitative framework for optimizing the heat treatment parameters of GH4780 superalloys in advanced aero-engine and industrial gas turbine applications. By accurately predicting a critical threshold temperature of 1080 °C, the model enables precise control of alloy grain size, ensuring optimal mechanical properties and long-term operational safety under extreme service conditions.

## Figures and Tables

**Table 1:** Measured composition of the GH4780 superalloy (wt.%).

Co	Cr	W	Nb	Ta	Al + Ti	C + B + Zr	Si	Ni
17.99	21.27	1.72	0.63	0.93	3.00	0.073	0.053	Bal.

**Table 2:** Heat treatment procedures explored for the GH4780 superalloy.

No.	Solution Treatment	Aging Treatment
AT1	1020 °C/1 h (AC)	800 °C/8 h (AC)
AT2	1050 °C/1 h (AC)	800 °C/8 h (AC)
AT3	1080 °C/1 h (AC)	800 °C/8 h (AC)
AT4	1110 °C/1 h (AC)	800 °C/8 h (AC)
ST1	1020 °C/1 h (WQ)	-
ST2	1050 °C/1 h (WQ)	-
ST3	1080 °C/1 h (WQ)	-
ST4	1110 °C/1 h (WQ)	-

## Data Availability

The original contributions presented in this study are included in the article. Further inquiries can be directed to the corresponding authors.
